# Rapid Identification and Classification of *Listeria* spp. and Serotype Assignment of *Listeria monocytogenes* Using Fourier Transform-Infrared Spectroscopy and Artificial Neural Network Analysis

**DOI:** 10.1371/journal.pone.0143425

**Published:** 2015-11-23

**Authors:** K. F. Romanolo, L. Gorski, S. Wang, C. R. Lauzon

**Affiliations:** 1 Department of Biological Sciences, California State University East Bay, Hayward, CA, United States of America; 2 United States Department of Agriculture, Agricultural Research Service, Western Regional Research Center, Albany, CA, United States of America; 3 Bruker Optics Inc, Fremont, CA, United States of America; University of Illinois at Chicago College of Medicine, UNITED STATES

## Abstract

The use of Fourier Transform-Infrared Spectroscopy (FT-IR) in conjunction with Artificial Neural Network software NeuroDeveloper™ was examined for the rapid identification and classification of *Listeria* species and serotyping of *Listeria monocytogenes*. A spectral library was created for 245 strains of *Listeria* spp. to give a biochemical fingerprint from which identification of unknown samples were made. This technology was able to accurately distinguish the *Listeria* species with 99.03% accuracy. Eleven serotypes of *Listeria monocytogenes* including 1/2a, 1/2b, and 4b were identified with 96.58% accuracy. In addition, motile and non-motile forms of *Listeria* were used to create a more robust model for identification. FT-IR coupled with NeuroDeveloper™ appear to be a more accurate and economic choice for rapid identification of pathogenic *Listeria* spp. than current methods.

## Introduction

Annual predictions estimate approximately 1,600 invasive infections, 1,500 hospitalizations and 250 deaths caused by *Listeria* species [1]. Of those individuals with laboratory- confirmed listeriosis, there is a 94% hospitalization rate and 15.9% death rate [1]. While *Listeria* infections are not common *per se*, there is significant mortality associated with these infections. Data show that this pathogen affects specific groups typically; the elderly, immunocompromised individuals, and pregnant women. Risk of listeriosis in pregnant women is ten times higher than the general population and four times higher in people aged sixty-five years or older [2]. Therefore, rapid and accurate identification of *Listeria* infections is mandated.

Two species of *Listeria*, *L*. *monocytogenes* and *L*.*ivanovii* are associated with listeriosis, which has an incubation period of 1–90 days, with symptoms including diarrhea, upset stomach, fever, chills, stiff neck, confusion, and muscle aches [3]. Listeriosis has two clinical manifestations: sepsis and meningitis [4] and is diagnosed when monocytosis is observed in both cerebrospinal fluid and peripheral blood [4]. Most cases are caused by *L*. *monocytogenes* of the serotypes 1/2a, 1/2b, 1/2c and 4b [5,6].

Current methods to speciate *Listeria* isolates involve classical microbiological culture, and PCR-based methods. Further characterization to the serotype level is done by agglutination, ELISA, and/or PCR methods [7,8]. Taken together, and depending on the techniques used, these identification and subtyping techniques can take from several hours to several days. A method that combines speciation and serotyping that occurs in a short time frame could be very useful during outbreak and traceback investigations.

Fourier-Transform Infrared Spectroscopy (FT-IR) is a powerful tool that has proven effective in identifying intact bacterial cells [9–11]. FT-IR analyzes the chemical bonding in the total biochemical composition of the cell including proteins, fatty acids, carbohydrates, nucleic acids, and lipopolysacharides [12]. Different strains of bacteria have unique, reproducible molecular fingerprints from which identification can be made from the slight changes in biochemical composition from species to species, even on the strain level [12].

Several studies report the ability of FT-IR to accurately identify bacterial species [13–17]. For example, FT-IR was used to differentiate *E*. *coli* from other pathogenic bacteria inoculated into apple juice [14]. Using soft independent modeling of class analogy (SIMCA) for chemometrics analysis, *E*. *coli* O157:H7 ATCC 35150 was differentiated from *E*. *coli* ATCC 25522 at an 82% confidence level [14]. Maquelin (2003) compared 89 bacterial strains and 32 yeast strains using FT-IR spectroscopy with 98.3% accurate identification [15]. Lopes (2013) showed that FT-IR was able to predict strains of *Streptococcus pneumoniae* serotype with 100% identification [16].

*Listeria* species have also been identified using FT-IR technology [17–20]. These reports all show correct identifications in the 90% or higher range. For example, Davis and Mauer (2011) showed a 96.6% correct identification rate using thirty different strains of *Listeria* comprising four different serotypes. The limiting factors in using FT-IR to speciate and/or serotype *Listeria* are data interpretation and cost. The development of artificial neural network software linked to FT-IR machines allows the creation of a robust database that defines *Listeria* species and serotypes that would be transferrable to other FT-IR devices. With the development of this database via the neural network a user could add bacterial sample to an FT-IR device containing the database definitions and allow the software to determine the species and/or the serotype. The present study describes improved efficacy and value of using FT-IR to identify *Listeria* spp. through the addition of artificial neural network software analysis. We reveal differentiatiation of 6 *Listeria* species and 11 different serotypes from 245 *Listeria* isolates.

## Materials and Methods

### Sample Preparation

Strains used in this study are shown in S1 Table. Individual *Listeria* spp. grown previously on a Tryticase Soy Agar (TSA, Becton-Dickinson-BBL, Franklin Lakes, NJ) were subcultured into 7mL Tryptic Soy Broth (TSB), and shaken at 150 rpm for 16 h at 37°C or 30°C. Aliquots of 1.5 mL of culture were subsequently placed into wells of a 2 mL 96-well microtitre plate (Eppendorf AG, Hamburg, Germany) and sealed with an aluminum plate cover (Excel Scientific, Inc. Victorville, CA). Each plate was centrifuged at 3000x rfc for 20 min at RT. Supernatants were removed and each pellet was washed with 0.85% saline solution and centrifuged again. Supernatants were removed and each pellet was resuspended in 100 μL of sterile dH_2_O and transferred into a 300 μL 96-well ELISA plate (Corning Incorporated, Corning, NY). Optical density (A_630_) readings were measured using a BioTek Synergy HT plate reader (BioTek, Winooski, VT), and samples were adjusted to an A_630_ target of 1.5–2.0. Five μL of each sample were spotted, in triplicate, onto a 384-well ZnSe plate (Bruker Ettlingen, Germany). Each plate was placed into a dry oven at 40°C and dried for 20 min. Plates were then placed on a heat block to 90°C for 10 min to kill the bacteria. After heating, spectra were acquired using a Bruker Optics FT-IR Spectrometer and HTS-XT Module (Bruker) in conjunction with Opus Lab (Version 7.2, Bruker) and NeuroDeveloper™ Software (Version 2.5b, Synthon Software).

### NeuroDeveloper Software Parameters

NeuroDeveloper software uses a series of techniques to simplify the large amounts of data found within a single spectrum. The first technique used is referred to as smoothing and averaging. A first derivative combined with Savitsky-Golay smoothing creates a spectrum that is easier to interpret. A spectrum containing 1763 points was averaged insofar that every five points were averaged into a single one. This reduced the total number of points in the spectra. Next, vector normalization was computed using the entire range of the spectra. This information was used to process spectra with different intensities or absorbance values to make them comparable. The vector-normalization calculates the vector norm of spectra or a subset of wavelengths. Lastly, unique points were used for creation of features. Features are independent wavelengths that create a unique identification. We used a Covar selection with 100 points. All these processes were executed within a designated spectral window. Our parameters were from x-minimum to 1800 cm^-1^, 2800 cm^-1^ to 3100 cm^-1^. The Artificial neural network net consists of three layers including an input layer, hidden layer and output layer. The input layer consisted of 100 neurons and 100 activation function neurons available. The hidden layer was comprised of 1 Neuron and a logistic activation function. The output layer consists of 2 neurons, a logistic activation function.

### *Listeria* species and serotype assignments

A three color rating system in NeuroDeveloper™ Software was used for *Listeria* species and serotype assignments to construct a database for comparison. A green dot indicated that 3/3 replicate scans matched to a correct species or serogroup identity. A purple diamond indicated that 2/3 replicate scans matched to a correct species or serogroup identity and lastly a red triangle indicate that only 1/3 or 0/3 replicate scans matched to a correct identity or serogroup identity. A 3/3 or 2/3 was considered to be a positive identification.

## Results

**Fig 1:**
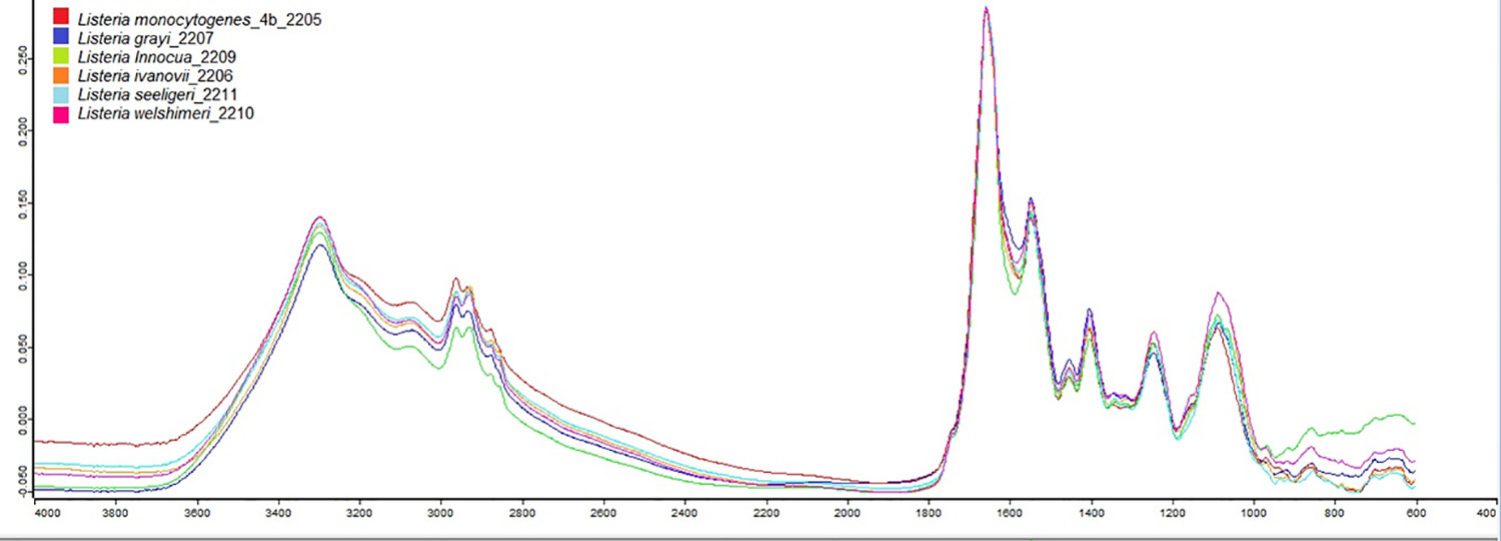
Raw FT-IR data of six *Listeria* species. *L*. *monocytogenes*, *L*. *grayi*, *L*. *innocua*, *L*. *ivanovii*, *L*. *seeligeri*, *L*. *welshemeri*.

A total of 245 *Listeria* isolates were used in this study and included six *Listeria* species: *L*.*grayi*, *L*. *innocua*, *L*. *ivanovii*, *L*. *monocytogenes*, *L*. *seeligeri*, *and L*. *welshimeri* (Table 1). Fig 1 shows the spectral profiles of all six *Listeria* species. Definitive assignments were detected in this *Listeria* species model by the ANN. Of 1,274 total spectra, positive identification (3/3 or 2/3) occurred approximately 99% (99.03), of the time. The ANN failed to give an assignment 3.30% of the time.

**Table 1:** *Listeria* species distribution and percentage of correctly identified spectra. Due to *Listeria monocytogenes*’ role in foodborne illness, there are a disproportional number of these isolates tested.

*Listeria* Species	N Isolates	Correct Identification
*Listeria monocytogenes*	206	99.91%
*Listeria innocua*	17	100.0%
*Listeria seeligeri*	10	54.17%
*Listeria ivanovii*	6	100.0%
*Listeria grayi*	3	100.0%
*Listeria welshimeri*	3	100.0%
**Total**	**245**	**99.13%**

**Fig 2:**
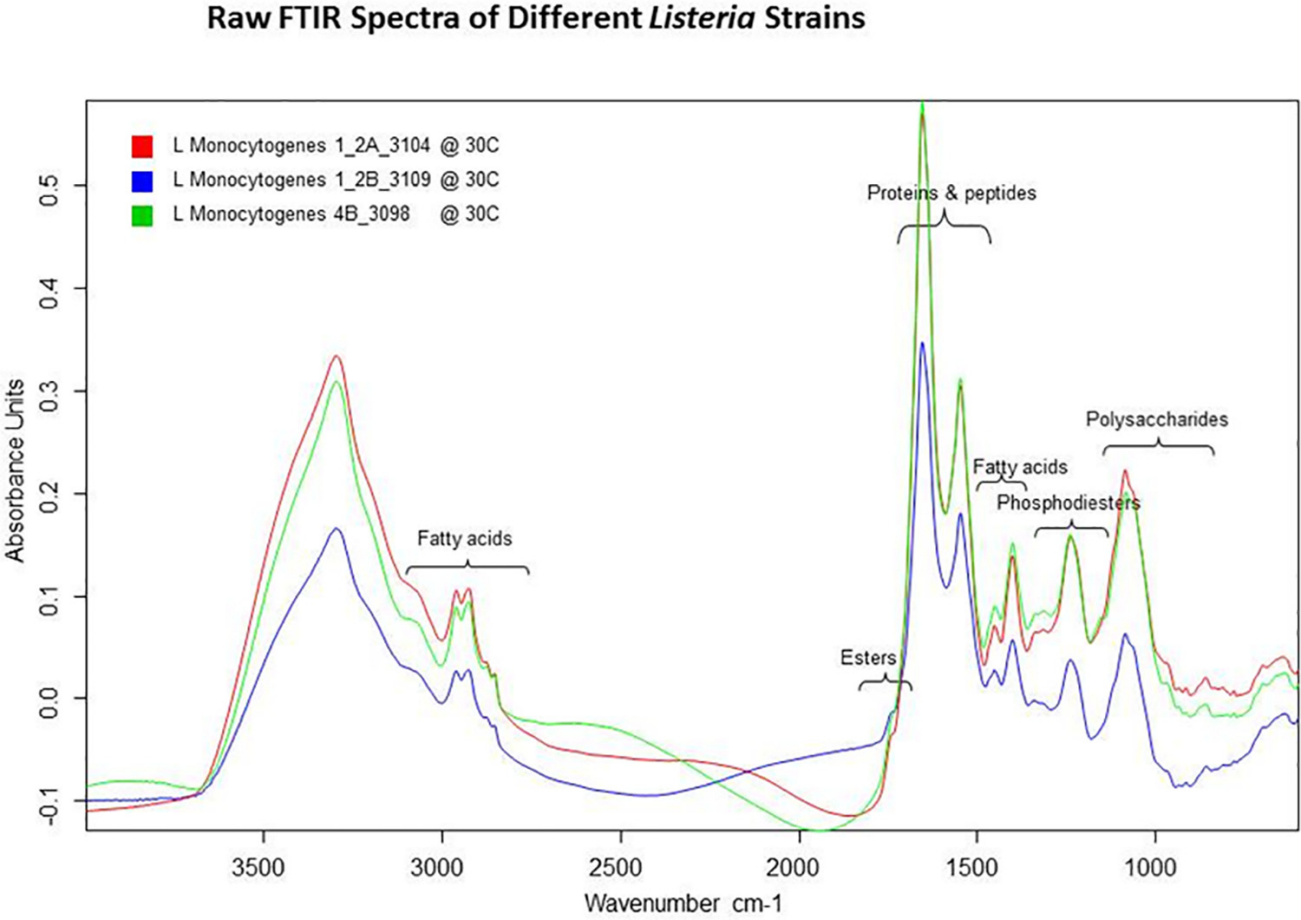
Raw FT-IR data showing three serogroups of *Listeria monocytogenes*. All strains were grown at 30°C. The spectral window shown is from x-minimum to 4,000 cm^-1^.

Creation of the *Listeria monocytogenes* serogroup model involved 1,023 raw spectra. A total of 206 different *Listeria monocytogenes* isolates were used in creating the serotype spectral library. *Listeria monocytogenes* serotypes used include: 1/2a, 1/2b, 1/2c, 3a, 3b, 3c, 4a, 4b, 4c, 4d, 4e. Correct identification occurred 96.58% of the time. Incorrect identifications occurred 3.42% of the time. The ANN failed to produce a serotype assignment 9.64% of the time. Fig 2 shows the spectral profiles of three *Listeria monocytogenes* 1/2a, 1/2b, 4b; the serotypes most associated with listeriosis in humans. Table 2 shows the correct identification percentage of each serotype considered in this model in the column labeled ANN 1.

**Table 2:** Eleven serovars of *Listeria monocytogenes* were used to form multiple artificial neural networks. Percentages show correct identification. Groupings based on somatic O antigens and trends in public safety.

*Serovar*	*N Isolates*	*ANN 1*	*ANN 2*	*ANN 3*	*ANN4*	*ANN5*
1/2a	49	94.89%	87.90%	93.68%	-	-
1/2b	46	92.83%	84.90%	95.03%	-	-
1/2c	14	82.43%	-	0%	-	-
3a	3	88.0%	-	-	100.0%	-
3b	3	0.0%	-	-	100.0%	-
3c	3	100.0%	-	-	100.0%	-
4a	2	100.0%	-	-	-	0%
4b	72	99.72%	98.40%	-	-	99.69%
4c	7	100.0%	-	-	-	100.0%
4d	3	100.0%	-	-	-	55.55%
4e	5	100.0%	-	-	-	50.0%
**Total**	**206**	**96.58%**	**90.25%**	**92.01%**	**100.0%**	**99.13%**

Four additional artificial neural networks were created for the analysis of *L*. *monocytogenes*. These included the 1/2 group (I, II O-Antigens), 3 group (II, IV O-Antigens), 4 group (V, VI, VII, IX O-Antigens) and most common foodborne *Listeria monocytogenes* serovars (1/2a, 1/2b, 4b). Table 2 shows the percentage of correctly identified spectra within each of these additional neural networks. Correct identification of serotypes depended on the make-up of the ANN. When only 1/2a, 1/2b, and 4b were compared (ANN 2) the percentage of correct identification of serotype 4b strains was over 98%, but the ability to discern between 1/2a and 1/2b dropped compared to ANN 1 indicating that serotype 4b was very easily distinguishable by the software, but less so between 1/2a and 1/2b. Therefore, we created ANNs based only on the serotype of the 1/2 strains (ANN 3), serotype 3 strains (ANN 4), and serotype 4 strains (ANN 5). In comparing only the serotype 1/2 strains the ANN could not distinguish 1/2c strains (Table 2). Among only serotype 3 strains the ANN could distinguish all of the limited number of strains tested. The results varied among serotype 4 strains, with nearly 100% accuracy for 4b and 4c strains, approximately 50% accuracy in identifying serotype 4d and 4e strains, and 0% accuracy in identifying the serotype 4a strains.

## Discussion

We found that FT-IR technology can be used to accurately distinguish several different *Listeria* species with 99.03% accuracy. Eleven serotypes of *Listeria monocytogenes* including 1/2a, 1/2b, and 4b were identified with 96.58% accuracy. Thus, we have built on existing studies through the expansion of the number of species, strains and serotypes from a greater variety of sources including those from clinical, environmental, and veterinary sources and/or from foods directly associated with outbreaks. Our project differs from previous studies because of the use of Artificial Neural Network for chemometric analysis of *Listeria* spp. and *Listeria monocytogenes*. Multiple neural networks were developed for identification of *Listeria monocytogenes* which shows that the serotypes included within the network can influence the percentage of correct identification. This network improves and learns with the addition of new data, rather than a static decision tree.

FT-IR spectra from whole bacterial cells are broad and overlapping due to the contribution of all biomolecules present, and therefore, single components cannot be identified [12]. This is not an uncommon event in chemical and biochemical analyses associated with bacterial species identification and differentiation. As a result, the use of FT-IR analysis for bacterial identification relies on software, such as ANN, to analyze raw data that provide a chemometric that is used to make identifications. ANN works well for managing overlapping data because it is an advanced multivariate data processing method of pattern analysis where large amounts of information are analyzed by training the data in a pattern recognition algorithm to recognize the particular combination of variables in a subset of data [21]. Classifications are made by the algorithm searching the combination of variables [21]. In the creation of the neural network, one needs to divide pre-defined, well characterized data. In our study we used ANN software to select one hundred features each for two different *Listeria* libraries. We set out to create a library that would define the group of *Listeria* species, and then created another library to differentiate strains of *L*. *monocytogenes* serotypes. In creating the model, the software used 80% of the spectra to create the definitions for the differentiation of the strains. The remaining 20% were treated as unknowns and were used to test the model. Therefore, the software had a built- in test for accuracy. The result provided a comparison library against which other FT-IR spectra from bacterial cells treated in the same fashion can be compared.

A limitation of FT-IR technology is that mixed cultures cannot be used. The single spectra that would result from a mixed culture would combine the biomolecules present from each of the microorganisms. Single organisms would not be able to be extracted from this spectra. Thus, single cultures are mandated in bacterial FT-IR methods. A perceived limitation is that there must be a certain level of expertise needed to use FT-IR technology; however, once the definition is created for the network, the user can simply place unknowns against our *Listeria* library and obtain identifications with the use of the NeuroDeveloper™ software. The output of the software is a three color rating system discussed in the materials section which leaves little interpretation on the side of the user. Lastly, cost of this method is inexpensive outside the initial investment of the instrument. In this study, we use Bruker Optics FT-IR Spectrometer and HTS-XT Module. The module is a plate reader platform for the ability read many spectra in a single run. Portable, single-sample FT-IR platforms are available, which may be able to reduce cost for individuals looking to adapt this platform at reduced cost.

We selected to use ANN in this study because it has been shown to be superior to Principal Component Analysis (PCA) and Hierarchical Cluster Analysis (HCA) for the discrimination of bacteria [12]. The advantage that ANN has over other chemometric analyses is that it is a “decision network” that becomes more robust with the inclusion of more data. In an outbreak situation, a particular strain’s spectral profile can be quickly added to the network. This would then influence the network to modify the definition of that particular serotype that would lead to a heightened ability to correctly identify unknown isolates.

The data from our multiple neural networks created show that the percentage of correct identification for a single serovar was influenced by what other serovars were in that neural network. For example, *Listeria monocytogenes* 3b had 100.0% correct identification within the 3 group (II, IV O-Antigens) however; in a network containing all *Listeria* serotypes, 3b was not correctly identified. All other serovars in that network saw correct identification percentages greater than 80%. The opposite was true for serovars 4d and 4e. In ANN 1, which contained data for all serovars, 100.0% correct identification occurred, whereas in ANN 5 containing 4 groups (V, VI, VII, IX O-Antigens) correct identity dropped to near 50%. Serovar 4b was successfully identified with greater than 98% accuracy in each network. It is likely that this high percentage was influenced by the large sample size of 72 isolates. Many serovars we tested had sample sizes less than ten isolates (i.e., 3a, 3b, 3c, 4a, 4c, 4d, 4e) because they are less frequently found in foodborne outbreaks and as a consequence are less in number in the USDA ARS isolate library. We feel that we can improve accuracy afforded by the use of ANN by increasing the sample size of these isolates although at this time, and in part, is a speculative notion. Isolates with high representation in our library included *Listeria monocytogenes* 1/2a, 1/2b and 4b. Each of these serovars had correct identities of 85% or higher in each of the artificial neural networks which demonstrates that our system can distinguish clinically- relevant serotypes, with ANN 1 and 2 as the most accurate of the networks created.

The power of this technology to accurately distinguish the *Listeria* species with 99.03% accuracy and serotypes of *Listeria monocytogenes* with 96.58% accuracy support the introduction of FT-IR coupled with NeuroDeveloper™ to food safety agencies. In addition, this approach would save time and money associated with outbreaks. Future plans include the differentiation of additional subtypes of *L*. *monocytogenes* to expand our library and identification capabilities.

## Supporting Information

S1 Table *Listeria* spp. strains used in this study. (DOCX)
