# Supplementary material for: Rapid Identification and Classification of Listeria spp. and Serotype Assignment of Listeria monocytogenes Using Fourier Transform-Infrared Spectroscopy and Artificial Neural Network Analysis
Source: PLoS One. 2015 Nov 23;10(11):e0143425. doi: 10.1371/journal.pone.0143425 (PMC4658148; doi:10.1371/journal.pone.0143425)
Supplement: S1 Table — (DOCX) [file pone.0143425.s001.docx]

**S1 Table. *Listeria* spp. strains used in this study.**

| **STRAIN** | **SPECIES** | **SEROTYPE** | **ATCC** | **DESCRIPTION** |
| --- | --- | --- | --- | --- |
| RM2207 | *Listeria grayi* |  | ATCC 19120 | Veterinary |
| RM2208 | *Listeria grayi* |  | ATCC 25401 | Food Source |
| RM 3344 | *Listeria grayi* |  |  | Food Source |
| RM 3343 | *Listeria innocua* | 6b | ATCC 33091 | Human |
| RM2209 | *Listeria innocua* | 6a | ATCC 33090 | Veterinary |
| RM 3304 | *Listeria innocua* | 4b |  | Environmental |
| RM 3313 | *Listeria innocua* | 4b |  | Food Source |
| RM 3315 | *Listeria innocua* | 4b |  | Environmental |
| RM2213 | *Listeria innocua* |  |  | Food Source |
| RM 2438 | *Listeria innocua* |  | ATCC 51742 | Food Source |
| RM 2443 | *Listeria innocua* |  |  | Food Source |
| RM 2444 | *Listeria innocua* |  |  | Food Source |
| RM 2445 | *Listeria innocua* |  |  | Food Source |
| RM 2446 | *Listeria innocua* |  |  | Food Source |
| RM3007 | *Listeria innocua* |  |  | Environmental |
| RM 3095 | *Listeria innocua* |  | ATCC 51742 | Food Source |
| RM 3318 | *Listeria innocua* |  |  | Food Source |
| RM 2442 | *Listeria innocua* |  |  | Food Source |
| RM 3319 | *Listeria innouca* |  |  | Food Source |
| RM 3320 | *Listeria innouca* |  |  | Food Source |
| RM2206 | *Listeria ivanovii* |  | ATCC 19119 | Veterinary |
| RM 3096 | *Listeria ivanovii* |  |  | Undetermined |
| RM 3325 | *Listeria ivanovii* |  |  | Food Source |
| RM 3326 | *Listeria ivanovii* |  |  | Food Source |
| RM 3345 | *Listeria ivanovii* |  |  | Veterinary |
| RM 3346 | *Listeria ivanovii londoniensis* |  | ATCC 49953 | Veterinary |
| RM2203 | *Listeria monocytogenes* | Unknown | ATCC 15313 | Veterinary |
| RM2204 | *Listeria monocytogenes* | Unknown | ATCC 19112 | Human |
| RM2218 | *Listeria monocytogenes* | 4e |  | Food Source |
| RM3820 | *Listeria monocytogenes* | 4e |  | Food Source |
| RM3821 | *Listeria monocytogenes* | 4e |  | Food Source |
| RM3822 | *Listeria monocytogenes* | 4e |  | Human |
| RM3823 | *Listeria monocytogenes* | 4e |  | Human |
| RM3025 | *Listeria monocytogenes* | 4d |  | Human |
| RM 3108 | *Listeria monocytogenes* | 4d |  | Environmental |
| RM3869 | *Listeria monocytogenes* | 4d |  | Undetermined |
| RM3022 | *Listeria monocytogenes* | 4c |  | Food Source |
| RM3030 | *Listeria monocytogenes* | 4c |  | Veterinary |
| RM3868 | *Listeria monocytogenes* | 4c |  | Veterinary |
| RM3894 | *Listeria monocytogenes* | 4c |  | Veterinary |
| RM3899 | *Listeria monocytogenes* | 4c |  | Veterinary |
| RM3901 | *Listeria monocytogenes* | 4c |  | Veterinary |
| RM3905 | *Listeria monocytogenes* | 4c |  | Veterinary |
| RM2199 | *Listeria monocytogenes* | 4b |  | Human |
| RM2200 | *Listeria monocytogenes* | 4b |  | Food Source |
| RM2201 | *Listeria monocytogenes* | 4b |  | Undetermined |
| RM2205 | *Listeria monocytogenes* | 4b | ATCC 19115 | Human |
| RM2215 | *Listeria monocytogenes* | 4b |  | Food Source |
| RM 2387 | *Listeria monocytogenes* | 4b |  | Food Source |
| RM2983 | *Listeria monocytogenes* | 4b |  | Veterinary |
| RM2984 | *Listeria monocytogenes* | 4b |  | Veterinary |
| RM2986 | *Listeria monocytogenes* | 4b |  | Food Source |
| RM2987 | *Listeria monocytogenes* | 4b |  | Human |
| RM2988 | *Listeria monocytogenes* | 4b |  | Food Source |
| RM2992 | *Listeria monocytogenes* | 4b |  | Food Source |
| RM2996 | *Listeria monocytogenes* | 4b |  | Food Source |
| RM2997 | *Listeria monocytogenes* | 4b |  | Food Source |
| RM2998 | *Listeria monocytogenes* | 4b |  | Human |
| RM3004 | *Listeria monocytogenes* | 4b |  | Environmental |
| RM3013 | *Listeria monocytogenes* | 4b |  | Human |
| RM3028 | *Listeria monocytogenes* | 4b |  | Human |
| RM 3098 | *Listeria monocytogenes* | 4b |  | Food Source |
| RM 3099 | *Listeria monocytogenes* | 4b |  | Food Source |
| RM 3100 | *Listeria monocytogenes* | 4b |  | Food Source |
| RM 3101 | *Listeria monocytogenes* | 4b |  | Human |
| RM 3103 | *Listeria monocytogenes* | 4b |  | Veterinary |
| RM 3150 | *Listeria monocytogenes* | 4b |  | Human |
| RM 3151 | *Listeria monocytogenes* | 4b |  | Human |
| RM 3153 | *Listeria monocytogenes* | 4b |  | Food Source |
| RM 3154 | *Listeria monocytogenes* | 4b |  | Human |
| RM 3173 | *Listeria monocytogenes* | 4b |  | Veterinary |
| RM 3176 | *Listeria monocytogenes* | 4b |  | Food Source |
| RM 3177 | *Listeria monocytogenes* | 4b |  | Human |
| RM 3178 | *Listeria monocytogenes* | 4b |  | Human |
| RM 3179 | *Listeria monocytogenes* | 4b |  | Human |
| RM 3180 | *Listeria monocytogenes* | 4b |  | Food Source |
| RM 3181 | *Listeria monocytogenes* | 4b |  | Food Source |
| RM 3182 | *Listeria monocytogenes* | 4b |  | Food Source |
| RM 3183 | *Listeria monocytogenes* | 4b |  | Food Source |
| RM 3186 | *Listeria monocytogenes* | 4b |  | Food Source |
| RM 3187 | *Listeria monocytogenes* | 4b |  | Human |
| RM 3301 | *Listeria monocytogenes* | 4b |  | Environmental |
| RM 3302 | *Listeria monocytogenes* | 4b |  | Veterinary |
| RM 3305 | *Listeria monocytogenes* | 4b |  | Human |
| RM 3356 | *Listeria monocytogenes* | 4b |  | Food Source |
| RM 3357 | *Listeria monocytogenes* | 4b |  | Food Source |
| RM 3358 | *Listeria monocytogenes* | 4b |  | Food Source |
| RM 3359 | *Listeria monocytogenes* | 4b |  | Human |
| RM 3360 | *Listeria monocytogenes* | 4b |  | Human |
| RM3399 | *Listeria monocytogenes* | 4b |  | Human |
| RM3401 | *Listeria monocytogenes* | 4b |  | Human |
| RM3404 | *Listeria monocytogenes* | 4b |  | Food Source |
| RM3813 | *Listeria monocytogenes* | 4b |  | Human |
| RM3815 | *Listeria monocytogenes* | 4b |  | Food Source |
| RM3817 | *Listeria monocytogenes* | 4b |  | Food Source |
| RM3818 | *Listeria monocytogenes* | 4b |  | Food Source |
| RM3825 | *Listeria monocytogenes* | 4b |  | Veterinary |
| RM3847 | *Listeria monocytogenes* | 4b |  | Human |
| RM3849 | *Listeria monocytogenes* | 4b |  | Human |
| RM3856 | *Listeria monocytogenes* | 4b |  | Veterinary |
| RM3858 | *Listeria monocytogenes* | 4b |  | Food Source |
| RM3861 | *Listeria monocytogenes* | 4b |  | Veterinary |
| RM3863 | *Listeria monocytogenes* | 4b |  | Food Source |
| RM3865 | *Listeria monocytogenes* | 4b |  | Veterinary |
| RM3878 | *Listeria monocytogenes* | 4b |  | Veterinary |
| RM3879 | *Listeria monocytogenes* | 4b |  | Human |
| RM3880 | *Listeria monocytogenes* | 4b |  | Human |
| RM3881 | *Listeria monocytogenes* | 4b |  | Food Source |
| RM3882 | *Listeria monocytogenes* | 4b |  | Human |
| RM3889 | *Listeria monocytogenes* | 4b |  | Human |
| RM4513 | *Listeria monocytogenes* | 4b |  | Human |
| RM4515 | *Listeria monocytogenes* | 4b |  | Food Source |
| RM4516 | *Listeria monocytogenes* | 4b |  | Human |
| RM4522 | *Listeria monocytogenes* | 4b |  | Human |
| RM4528 | *Listeria monocytogenes* | 4b |  | Human |
| RM3396 | *Listeria monocytogenes* | 4a |  | Undetermined |
| RM3397 | *Listeria monocytogenes* | 4a |  | Human |
| RM3027 | *Listeria monocytogenes* | 3c |  | Food Source |
| RM 3159 | *Listeria monocytogenes* | 3c |  | Human |
| RM 3113 | *Listeria monocytogenes* | 3b |  | Environmental |
| RM 3121 | *Listeria monocytogenes* | 3b |  | Environmental |
| RM 3122 | *Listeria monocytogenes* | 3b |  | Environmental |
| RM3026 | *Listeria monocytogenes* | 3a |  | Food Source |
| RM 3120 | *Listeria monocytogenes* | 3a |  | Environmental |
| RM 3162 | *Listeria monocytogenes* | 3a |  | Human |
| RM2195 | *Listeria monocytogenes* | 1/2c |  | Undetermined |
| RM3000 | *Listeria monocytogenes* | 1/2c |  | Environmental |
| RM3014 | *Listeria monocytogenes* | 1/2c |  | Human |
| RM3017 | *Listeria monocytogenes* | 1/2c |  | Human |
| RM3018 | *Listeria monocytogenes* | 1/2c |  | Human |
| RM3019 | *Listeria monocytogenes* | 1/2c |  | Food Source |
| RM3020 | *Listeria monocytogenes* | 1/2c |  | Food Source |
| RM3021 | *Listeria monocytogenes* | 1/2c |  | Human |
| RM 3105 | *Listeria monocytogenes* | 1/2c |  | Environmental |
| RM 3314 | *Listeria monocytogenes* | 1/2c |  | Food Source |
| RM3395 | *Listeria monocytogenes* | 1/2c |  | Food Source |
| RM3400 | *Listeria monocytogenes* | 1/2c |  | Human |
| RM3829 | *Listeria monocytogenes* | 1/2c |  | Food Source |
| RM3839 | *Listeria monocytogenes* | 1/2c |  | Food Source |
| RM2216 | *Listeria monocytogenes* | 1/2b |  | Food Source |
| RM2217 | *Listeria monocytogenes* | 1/2b |  | Food Source |
| RM 2388 | *Listeria monocytogenes* | 1/2b |  | Food Source |
| RM2707 | *Listeria monocytogenes* | 1/2b |  | Food Source |
| RM2708 | *Listeria monocytogenes* | 1/2b |  | Food Source |
| RM2709 | *Listeria monocytogenes* | 1/2b |  | Food Source |
| RM2710 | *Listeria monocytogenes* | 1/2b |  | Food Source |
| RM2711 | *Listeria monocytogenes* | 1/2b |  | Food Source |
| RM2712 | *Listeria monocytogenes* | 1/2b |  | Food Source |
| RM2713 | *Listeria monocytogenes* | 1/2b |  | Food Source |
| RM2714 | *Listeria monocytogenes* | 1/2b |  | Food Source |
| RM2991 | *Listeria monocytogenes* | 1/2b |  | Veterinary |
| RM2995 | *Listeria monocytogenes* | 1/2b |  | Veterinary |
| RM3024 | *Listeria monocytogenes* | 1/2b |  | Undetermined |
| RM 3109 | *Listeria monocytogenes* | 1/2b |  | Environmental |
| RM 3115 | *Listeria monocytogenes* | 1/2b |  | Environmental |
| RM 3156 | *Listeria monocytogenes* | 1/2b |  | Human |
| RM 3157 | *Listeria monocytogenes* | 1/2b |  | Veterinary |
| RM 3188 | *Listeria monocytogenes* | 1/2b |  | Food Source |
| RM 3189 | *Listeria monocytogenes* | 1/2b |  | Human |
| RM 3307 | *Listeria monocytogenes* | 1/2b |  | Environmental |
| RM 3309 | *Listeria monocytogenes* | 1/2b |  | Food Source |
| RM 3310 | *Listeria monocytogenes* | 1/2b |  | Food Source |
| RM 3311 | *Listeria monocytogenes* | 1/2b |  | Environmental |
| RM 3312 | *Listeria monocytogenes* | 1/2b |  | Environmental |
| RM 3317 | *Listeria monocytogenes* | 1/2b |  | Food Source |
| RM 3350 | *Listeria monocytogenes* | 1/2b |  | Food Source |
| RM3816 | *Listeria monocytogenes* | 1/2b |  | Food Source |
| RM3828 | *Listeria monocytogenes* | 1/2b |  | Food Source |
| RM3830 | *Listeria monocytogenes* | 1/2b |  | Food Source |
| RM3832 | *Listeria monocytogenes* | 1/2b |  | Food Source |
| RM3833 | *Listeria monocytogenes* | 1/2b |  | Food Source |
| RM3837 | *Listeria monocytogenes* | 1/2b |  | Food Source |
| RM3838 | *Listeria monocytogenes* | 1/2b |  | Food Source |
| RM3843 | *Listeria monocytogenes* | 1/2b |  | Food Source |
| RM3846 | *Listeria monocytogenes* | 1/2b |  | Food Source |
| RM3851 | *Listeria monocytogenes* | 1/2b |  | Veterinary |
| RM3853 | *Listeria monocytogenes* | 1/2b |  | Veterinary |
| RM3871 | *Listeria monocytogenes* | 1/2b |  | Environmental |
| RM3874 | *Listeria monocytogenes* | 1/2b |  | Veterinary |
| RM3877 | *Listeria monocytogenes* | 1/2b |  | Veterinary |
| RM3883 | *Listeria monocytogenes* | 1/2b |  | Environmental |
| RM3885 | *Listeria monocytogenes* | 1/2b |  | Food Source |
| RM3887 | *Listeria monocytogenes* | 1/2b |  | Food Source |
| RM3888 | *Listeria monocytogenes* | 1/2b |  | Food Source |
| RM3893 | *Listeria monocytogenes* | 1/2b |  | Veterinary |
| RM 2390 | *Listeria monocytogenes* | 1/2a |  | Undetermined |
| RM2985 | *Listeria monocytogenes* | 1/2a |  | Food Source |
| RM2989 | *Listeria monocytogenes* | 1/2a |  | Food Source |
| RM2990 | *Listeria monocytogenes* | 1/2a |  | Food Source |
| RM3015 | *Listeria monocytogenes* | 1/2a |  | Food Source |
| RM3023 | *Listeria monocytogenes* | 1/2a |  | Food Source |
| RM3029 | *Listeria monocytogenes* | 1/2a |  | Food Source |
| RM 3102 | *Listeria monocytogenes* | 1/2a |  | Veterinary |
| RM 3104 | *Listeria monocytogenes* | 1/2a |  | Environmental |
| RM 3106 | *Listeria monocytogenes* | 1/2a |  | Environmental |
| RM 3152 | *Listeria monocytogenes* | 1/2a |  | Veterinary |
| RM 3160 | *Listeria monocytogenes* | 1/2a |  | Human |
| RM 3175 | *Listeria monocytogenes* | 1/2a |  | Food Source |
| RM 3184 | *Listeria monocytogenes* | 1/2a |  | Food Source |
| RM 3185 | *Listeria monocytogenes* | 1/2a |  | Food Source |
| RM 3300 | *Listeria monocytogenes* | 1/2a |  | Environmental |
| RM 3303 | *Listeria monocytogenes* | 1/2a |  | Environmental |
| RM 3306 | *Listeria monocytogenes* | 1/2a |  | Environmental |
| RM 3316 | *Listeria monocytogenes* | 1/2a |  | Environmental |
| RM 3349 | *Listeria monocytogenes* | 1/2a |  | Food Source |
| RM 3352 | *Listeria monocytogenes* | 1/2a |  | Environmental |
| RM 3354 | *Listeria monocytogenes* | 1/2a |  | Environmental |
| RM 3355 | *Listeria monocytogenes* | 1/2a |  | Environmental |
| RM3398 | *Listeria monocytogenes* | 1/2a |  | Environmental |
| RM3814 | *Listeria monocytogenes* | 1/2a |  | Food Source |
| RM3819 | *Listeria monocytogenes* | 1/2a |  | Food Source |
| RM3824 | *Listeria monocytogenes* | 1/2a |  | Veterinary |
| RM3826 | *Listeria monocytogenes* | 1/2a |  | Veterinary |
| RM3827 | *Listeria monocytogenes* | 1/2a |  | Food Source |
| RM3831 | *Listeria monocytogenes* | 1/2a |  | Food Source |
| RM3834 | *Listeria monocytogenes* | 1/2a |  | Food Source |
| RM3835 | *Listeria monocytogenes* | 1/2a |  | Food Source |
| RM3840 | *Listeria monocytogenes* | 1/2a |  | Food Source |
| RM3841 | *Listeria monocytogenes* | 1/2a |  | Food Source |
| RM3842 | *Listeria monocytogenes* | 1/2a |  | Food Source |
| RM3844 | *Listeria monocytogenes* | 1/2a |  | Food Source |
| RM3850 | *Listeria monocytogenes* | 1/2a |  | Veterinary |
| RM3852 | *Listeria monocytogenes* | 1/2a |  | Veterinary |
| RM3864 | *Listeria monocytogenes* | 1/2a |  | Veterinary |
| RM3866 | *Listeria monocytogenes* | 1/2a |  | Food Source |
| RM3875 | *Listeria monocytogenes* | 1/2a |  | Veterinary |
| RM3876 | *Listeria monocytogenes* | 1/2a |  | Food Source |
| RM3884 | *Listeria monocytogenes* | 1/2a |  | Veterinary |
| RM3890 | *Listeria monocytogenes* | 1/2a |  | Environmental |
| RM3891 | *Listeria monocytogenes* | 1/2a |  | Undetermined |
| RM4498 | *Listeria monocytogenes* | 1/2a |  | Food Source |
| RM4512 | *Listeria monocytogenes* | 1/2a |  | Food Source |
| RM4514 | *Listeria monocytogenes* | 1/2a |  | Food Source |
| RM4517 | *Listeria monocytogenes* | 1/2a |  | Human |
| RM 3335 | *Listeria monocytogenes* |  |  | Food Source |
| RM 3366 | *Listeria monocytogenes* |  |  | Human |
| RM4497 | *Listeria monocytogenes* |  |  | Human |
| RM4518 | *Listeria monocytogenes* |  |  | Human |
| RM4558 | *Listeria monocytogenes* |  |  | Human |
| RM 3097 | *Listeria seeligeri* |  | ATCC 35967 | Environmental |
| RM 3341 | *Listeria seeligeri* |  | ATCC 51334 | Veterinary |
| RM 3321 | *Listeria seeligeri* |  |  | Food Source |
| RM2211 | *Listeria seeligeri* |  | ATCC 35867 | Environmental |
| RM3008 | *Listeria seeligeri* |  |  | Environmental |
| RM3009 | *Listeria seeligeri* |  |  | Environmental |
| RM3010 | *Listeria seeligeri* |  |  | Environmental |
| RM3011 | *Listeria seeligeri* |  |  | Environmental |
| RM3012 | *Listeria seeligeri* |  |  | Environmental |
| RM 3342 | *Listeria seeligeri* |  |  | Food Source |
| RM2210 | *Listeria welshimeri* | 6b | ATCC 35897 | Environmental |
| RM 3323 | *Listeria welshimeri* |  |  | Food Source |
| RM 3324 | *Listeria welshimeri* |  |  | Food Source |
